# Toll-like receptor dual-acting agonists are potent inducers of PBMC-produced cytokines that inhibit hepatitis B virus production in primary human hepatocytes

**DOI:** 10.1038/s41598-020-69614-7

**Published:** 2020-07-29

**Authors:** Vaclav Janovec, Jan Hodek, Kamila Clarova, Tomas Hofman, Pavel Dostalik, Jiri Fronek, Jaroslav Chlupac, Laurence Chaperot, Sarah Durand, Thomas F. Baumert, Iva Pichova, Barbora Lubyova, Ivan Hirsch, Jan Weber

**Affiliations:** 1 Department of Genetics and Microbiology, Faculty of Science, Charles University, BIOCEV, 25150 Vestec, Czech Republic; 2 IOCB & Gilead Research Center, Institute of Organic Chemistry and Biochemistry of the Czech Academy of Science, 16610 Prague, Czech Republic; 3 Transplantation Surgery Department, Institute for Clinical and Experimental Medicine, 14021 Prague, Czech Republic; 4 Department of Anatomy, Second Faculty of Medicine, Charles University, 15006 Prague, Czech Republic; 5 CNRS UMR5309, Inserm U1209, CHU Grenoble Alpes, IAB, EFS, Université Grenoble Alpes, 38000 Grenoble, France; 6 Inserm, Institut de Recherche Sur Les Maladies Virales Et Hepatiques UMRS 1110, Universite de Strasbourg, 67000 Strasbourg, France; 7 Pole Hepato-Digestif, Institut Hospitalo-Universitaire, Hopitaux Universitaires de Strasbourg, 67000 Strasbourg, France; 8 Institute of Molecular Genetics of the Czech Academy of Sciences, 14220 Prague, Czech Republic

**Keywords:** Drug discovery, Immunology, Diseases, Gastroenterology, Medical research, Pathogenesis

## Abstract

Recombinant interferon-α (IFN-α) treatment functionally cures chronic hepatitis B virus (HBV) infection in some individuals and suppresses virus replication in hepatocytes infected in vitro. We studied the antiviral effect of conditioned media (CM) from peripheral blood mononuclear cells (PBMCs) stimulated with agonists of Toll-like receptors (TLRs) 2, 7, 8 and 9. We found that CM from PBMCs stimulated with dual-acting TLR7/8 (R848) and TLR2/7 (CL413) agonists were more potent drivers of inhibition of HBe and HBs antigen secretion from HBV-infected primary human hepatocytes (PHH) than CM from PBMCs stimulated with single-acting TLR7 (CL264) or TLR9 (CpG-B) agonists. Inhibition of HBV in PHH did not correlate with the quantity of PBMC-produced IFN-α, but it was a complex function of multiple secreted cytokines. More importantly, we found that the CM that efficiently inhibited HBV production in freshly isolated PHH via various cytokine repertoires and mechanisms did not reduce covalently closed circular (ccc)DNA levels. We confirmed our data with a cell culture model based on HepG2-NTCP cells and the plasmacytoid dendritic cell line GEN2.2. Collectively, our data show the importance of dual-acting TLR agonists inducing broad cytokine repertoires. The development of poly-specific TLR agonists provides novel opportunities towards functional HBV cure.

## Introduction

Chronic infection with hepatitis B virus (HBV) is a major public health problem affecting approximately 250 million people worldwide. Despite a weak innate immune response to HBV approximately 90% of adults clear HBV, presumably via the induction of an effective CD8 + T cell response^1–3^. Treatment of chronic hepatitis B with nucleot(s)ide analogues inhibits the formation of new infectious viral particles but does not eliminate stable covalently closed circular DNA (cccDNA) in hepatocytes. Pegylated interferon α (IFN-α) treatment can be considered as an alternative therapy for people with mild-to-moderate chronic hepatitis B^4^. However, in addition to causing undesired side effects, IFN-α monotherapy leads to functional cure in less than 8% of people with chronic hepatitis B^4–7^.

Results from cell culture experiments have demonstrated that type I IFNs (IFN-I, IFN-α, β, ε, ω) as well as type III IFNs (IFN-III, IFN-λ1, 2, 3) affect HBV cccDNA either directly through epigenetic transcriptional silencing^8^ or by reducing its stability^9–11^. In HBV-infected hepatocytes, IFN-I induces hundreds of IFN-stimulated genes (ISGs) that restrict HBV infection at different levels^12^. IFN-α induces soluble factors that inhibit HBV entry into cells^13^, protein kinase R, which inhibits HBV protein translation^14^, and tetherin, which blocks release of HBV from infected hepatocytes^15^. A side-by-side comparison of a large panel of cytokines in vitro revealed that proinflammatory cytokines, such as tumor necrosis factor α (TNF-α), interleukin (IL)-1β and IL-6, are as efficient as IFNs at inhibiting HBV replication^16–18^. Thus, both IFNs and proinflammatory cytokines control HBV replication and contribute to HBV cure in different models^17,19^.

The lack of curative anti-HBV therapies highlights the potential importance of different immune-modulators and their agonists^20–24^. Among agonists of pattern recognizing receptors expressed in primary liver cells^25^, namely agonists of Toll-like receptors (TLRs) attracted interest because of their potency to induce IFNs and proinflammatory cytokines and chemokines in both hepatocytes and non-parenchymal cells^23,24^. Moreover, it was shown that GS-9620 (vesatolimod), an agonist of endosomal TLR7, which is preferentially expressed in plasmacytoid dendritic cells (pDCs)^26–30^ but not in primary hepatocytes (PHH)^23,31,32^, significantly reduced viremia and cccDNA expression, and led to functional cure in animal models^20–22^. A recent study showed that TLR1/2 and TLR3 ligands inhibit HBV replication in PHH, and that the same ligands also induce the production of antiviral cytokines in peripheral blood mononuclear cells (PBMCs)^23^. Another study showed that inhibition of HBV replication in PHH could be mediated by conditioned media (CM) from PBMCs stimulated with GS-9620^24^. IFN-I secreted by TLR7-agonist-stimulated PBMCs was identified as the major substance inhibiting HBV production without reducing cccDNA levels^24^.

Several studies demonstrated that combination of different TLR agonists or a single TLR agonist with other immune-modulators potentiated the immunotherapeutic effect^33,34^. However, the effect of poly-specific TLR agonists like recently developed TLR2/7 dual-acting agonist CL413 (Adilipolin), a chimeric molecule that co-activates the cell surface receptor TLR2 and the endosomal receptor TLR7^35^, on HBV infection was not elucidated. Here, we compared the antiviral effect of CM from PBMCs stimulated with dual-acting agonists with the effect of CM from PBMCs stimulated with agonists for single TLR. We found that CM from PBMC stimulated with a dual-acting agonist of TLR7/8 (R848) and TLR2/7 (CL413) were more potent drivers of inhibition of hepatitis *e* and *s* antigens (HBeAg and HBsAg) production from HBV-infected PHH than CM from PBMCs stimulated with agonists specific only for TLR7 (GS-9620, CL264) or TLR9 (CpG-A, CpG-B). Inhibition of HBV in PHH did not correlate with the level of PBMC-produced IFN-α, but it was a complex function of multiple secreted cytokines. We addressed the question whether CM, which efficiently inhibited the production of HBV in PHH via different repertoires of cytokines would also reduce the cccDNA levels.

## Results

**Figure 1:**
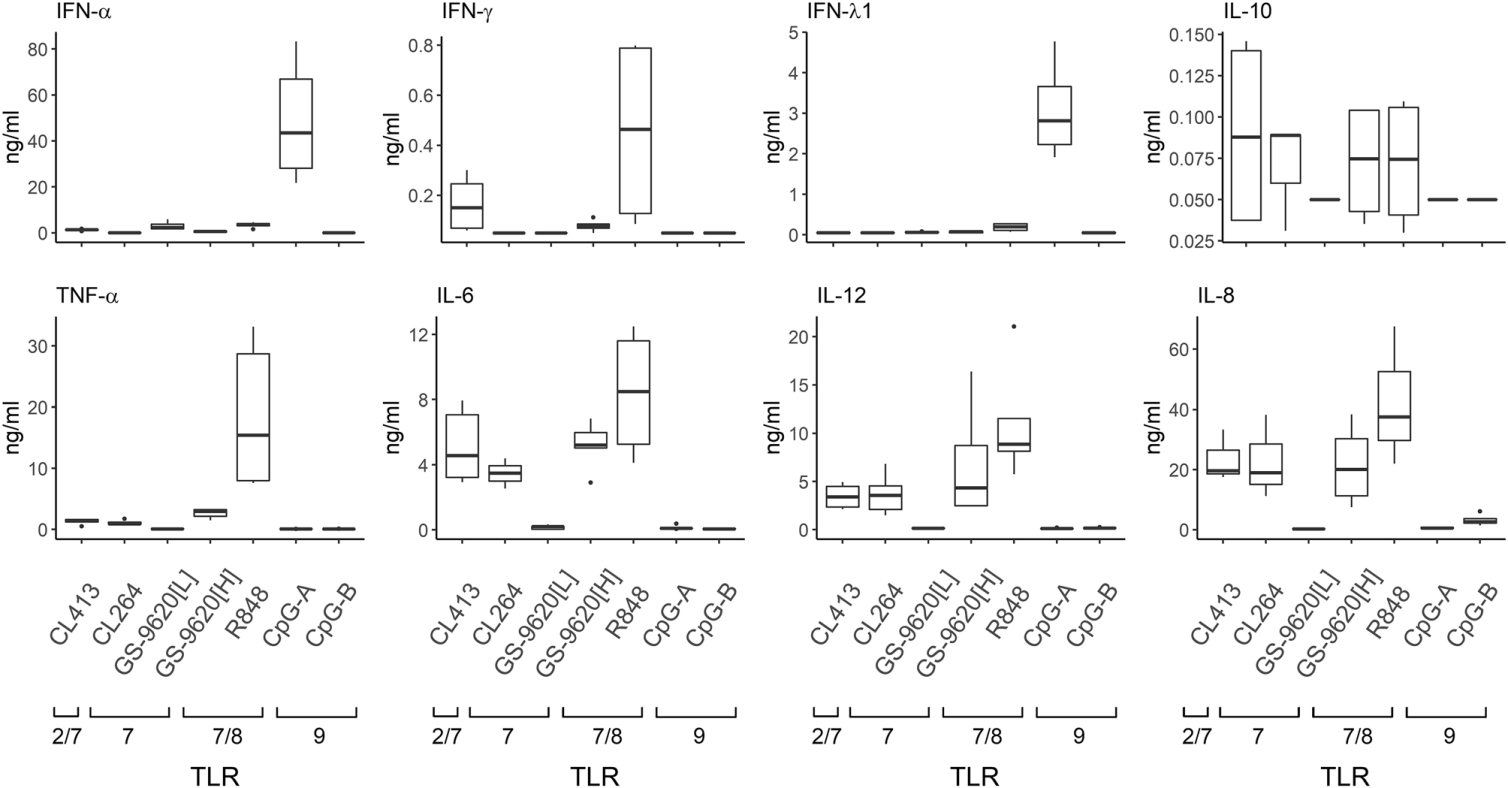
Cytokines secreted by PBMCs stimulated by different TLR2/7, TLR7, TLR7/8 and TLR9 agonists. PBMCs (N > 3) were stimulated with the TLR2/7 dual-agonist CL413 (5 µg/ml), the TLR7 agonists CL264 (5 µg/ml) and GS-9620[L] (50 nM), the TLR7/8 agonists GS-9620[H] (10 µM) and R848 (4 µg/ml), and the TLR9 agonist CpG-A (4 µg/ml) or CpG-B (4 µg/ml) for 16 h, and the cytokine levels were determined by ELISA. The data are shown as medians and interquartile ranges. See Supplementary Fig. S1 for logarithmic plot.

**Differential potency of TLR agonists in the induction of PBMC-secreted cytokines.** First, we determined the levels of selected cytokines secreted into supernatants (conditioned media, CM) of PBMCs stimulated for 16 h by different agonists of TLR7 (CL264-CM, GS-9620[L]-CM (50 nM)), TLR7/8 (R848-CM, GS-9620[H]-CM (10 µM)), TLR9 (CpG-A-CM, CpG-B-CM) and a TLR2/7 dual agonist (CL413-CM) (Fig. 1, linear plot, Supplementary Fig. S1, logarithmic plot). Two concentrations of GS-9620 were used: at a low concentration (GS-9620[L], 50 nM) it shows a high selectivity for activation of TLR7 over TLR8^36^, while a higher concentration (GS-9620[H], 10 µM) elicits combined TLR7 and TLR8 stimulation. Among the cytokines present in CM, we quantified those previously shown to regulate HBV replication, including type I, II and III IFNs (IFN-α, γ, λ); the proinflammatory cytokines TNF-α, IL-6 and IL-12; the chemokine IL-8; and the regulatory cytokine IL-10^8–11,13–15^. While IFN-α and IFN-λ1 were predominantly induced by CpG-A, the proinflammatory cytokines IFN-γ, TNF-α, IL-6, IL-8 and IL-12 were predominantly induced by R848. IL-6, IL-8 and IL-12 were also significantly stimulated by CL264-CM, GS-9620[H]-CM and CL413-CM. The latter agonists also stimulated production of the anti-inflammatory cytokine IL-10. Then, we determined by dynamic phospho-flow cytometry phosphorylation of the NF-ĸB p65 subunit in PBMCs exposed for 1 h to different TLR agonists (Supplementary Fig. S2)^37^. Stimulation for this time interval, which was insufficient for cytokine production, resulted in phosphorylation of p65 NF-ĸB in PBMCs exposed to dual-acting agonists R848 (20.3%), CL413 (20.8%) and GS-9620[H] (6.3%). In contrast, the single-acting agonists, GS-9620[L] (0.6%) and CpG-A (0.6%), did not induce the NF-ĸB p65 phosphorylation. In summary, PBMCs stimulated by different TLR2/7, TLR7, TLR7/8 and TLR9 agonists produced broad and variable repertoires of type I, II and III IFNs and proinflammatory cytokines.

**Figure 2:**
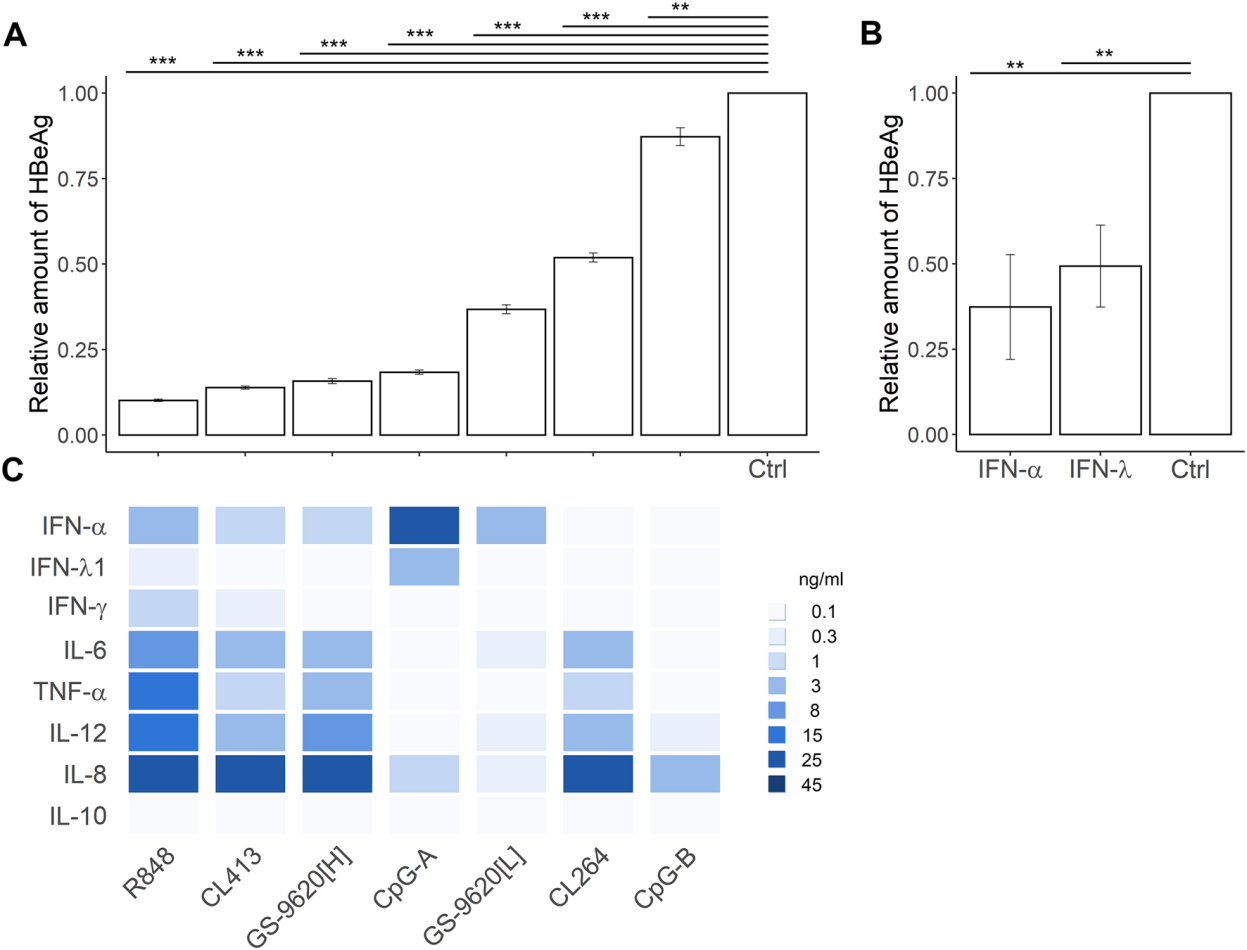
Inhibition of HBeAg production from HBV-infected PHH treated with PBMC CM. (**A**) A total of 65,000 PHH were infected with 500 viral genome equivalents (VGE) of HBV per cell and cultured for 3 days before conditioned medium (CM, diluted 1:10) was added. CM was derived from 3 × 10^6^ PBMCs per ml stimulated by agonists of TLR2/7 (CL413), TLR7 (CL264, GS-9620[L]), TLR7/8, (R848, GS-9620[H]), or TLR9 (CpG-A, CpG-B) for 16 h. CM was added again 6 days post-infection (DPI). Production of HBeAg was determined by ELISA 9 DPI and normalized to production by HBV-infected PHH in the absence of CM. The HBeAg data are shown as mean ± SEM from five independent experiments with PHH from three donors (N = 3). **p < 0.01, ***p < 0.001 pairwise Wilcoxon test. Kruskal–Wallis *p* < 2.2 × e^−16^. (B) HBV-infected PHH treated with 1,000 IU/ml of recombinant IFN-α-2a or IFN-λ3. The data are shown as mean ± SEM from three independent experiments with PHH from two donors (N = 2). **p < 0.01, Mann–Whitney–Wilcoxon pairwise test, *p* value adjusted by Benjamini–Hochberg (BH) method. (**C**) Quantity of cytokines in CM from stimulated PBMCs plotted as a heat diagram representing the median values that is shown in Fig. 1.

**HBV production in infected PHH is inhibited by exposure to CM from PBMCs stimulated with TLR2/7, TLR7, TLR7/8 and TLR9 agonists.** We examined the effect of CM from PBMCs stimulated with different agonists of TLR2/7, TLR7, TLR7/8 and TLR9 on HBeAg (Fig. 2A) and HBsAg (Supplementary Fig. S3) production from PHH infected with HBV from 3 to 9 days post-infection (DPI). None of PBMC CM affected PHH viability (Supplementary Table S1). Production of HBeAg was significantly inhibited by CM from PBMCs stimulated with R848 (by 89%, *p* = 5.70e−09), CL413 (by 85%, *p* = 5.70e−09), CpG-A (by 80%, *p* = 5.70e−09), GS-9620[H] (by 83%, *p* = 5.70e−09), GS9620[L] (by 59%, *p* = 5.70e−09), CL264 (by 42%, *p* = 5.70e−09), CpG-B (by 9%, *p* = 2.24e−03) (Fig. 2A, supplementary Table S2, for significance) and by recombinant IFN-α-2a and IFN-λ3 (Fig. 2B). Significantly higher inhibition of HBeAg was achieved with R848-CM, CL413-CM and GS-9620[H]-CM compared to GS-9620[L]-CM (Fig. 2C). Notably, significantly higher inhibition of HBeAg was achieved with GS-9620[H]-CM, which contained a lower quantity of IFN-α but higher levels of the proinflammatory cytokines IL-6, TNF-α and IL-12, than with GS-9620[L]-CM. Within the variable repertoires of IFNs and proinflammatory cytokines, the levels of IFN-α and IFN-λ1 and the levels of the proinflammatory cytokines and chemokines tested—IFN-γ, TNF-α, IL-6, IL-8 and IL-12—correlated across the agonists evaluated (R ≥ 0.7) (Supplementary Table S3). More importantly, HBeAg levels negatively correlated with the quantity of IFN-γ, TNF-α, IL-6, IL-8 and IL-12 (R ≥ 0.7). Taken together, analysis of HBeAg production revealed that inhibitory levels do not correlate with the quantity of secreted IFN-α when other antiviral cytokines like IL-6, TNF-α and IFN-γ are produced by PBMCs. Our data support a model where not a single cytokine, but a complex function of multiple PBMC-secreted cytokines is associated with CM-mediated HBV inhibition in PHH.

**Figure 3:**
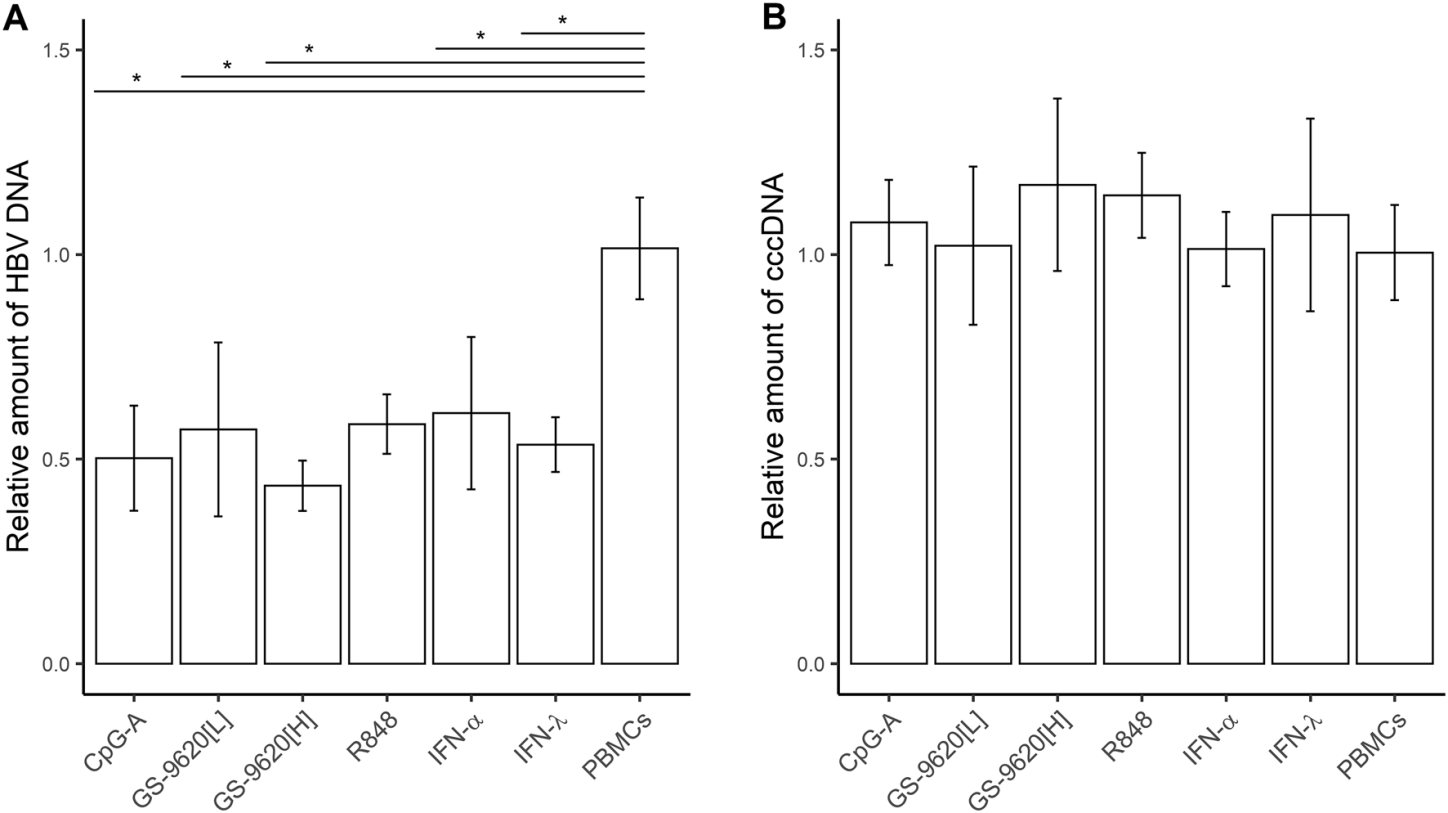
Reduction of total HBV DNA (**A**) but not cccDNA (**B**) in HBV-infected PHH by CM from TLR agonist-stimulated PBMCs. PHH were infected with HBV and cultured for 3 days followed by addition of CpG-A-CM, GS-9620[L]-CM, GS-9620[H]-CM or R848–CM (diluted 1:10) or 1,000 IU of IFN-α or IFN-λ. CM was added again 6 DPI. Cells were grown for 3 more days and the quantities of total HBV DNA and cccDNA were determined by qPCR. Data are shown as mean ± SEM with PHH from three donors (N = 3) for CpG-A-CM, GS-9620[L]-CM, and with PHH from one donor (N = 1) for GS-9620[H]-CM, R848-CM (two biological replicates) *p < 0.05, Dunn’s test, *p* value adjusted by Benjamini-Hochberg (BH) method.

**Total HBV DNA, but not cccDNA, in HBV-infected PHH is reduced by CMs from TLR2/7, TLR7, TLR7/8, and TLR9 agonist-stimulated PBMCs. **Treatment of freshly isolated HBV-infected PHH with CpG-A-CM, GS-9620[L]-CM, GS-9620[H]-CM or R848-CM or treatment with 1,000 IU of recombinant IFN-α or IFN-λ led to an approximately 50% reduction in intracellular HBV DNA levels (Fig. 3A). No decrease in cccDNA was detected in the same DNA samples from three PHH donors by qPCR using specific cccDNA primers (Kruskal–Wallis *p* = 0.443) (Fig. 3B). In addition, we used qPCR to evaluate the effect of the dual TLR agonists-induced R848-CM and GS-9620[H]-CM on HBV cccDNA in PHH from one donor. However, CM from PBMCs stimulated by these dual TLR agonists also did not reduce the cccDNA level. Moreover, we used droplet-digital (dd)PCR to verify the effect of TLR dual agonists, including GS-9620[H]-CM or R848-CM and CL413-CM, on cccDNA and to assess the quality of cccDNA sample preparation (Supplementary Table S4). Data obtained by ddPCR confirmed the importance of T5 exonuclease treatment and selection of cccDNA-specific primers. Collectively, our results suggest that none of the selected TLR agonists reduced cccDNA in our in vitro PHH culture system.

**Figure 4:**
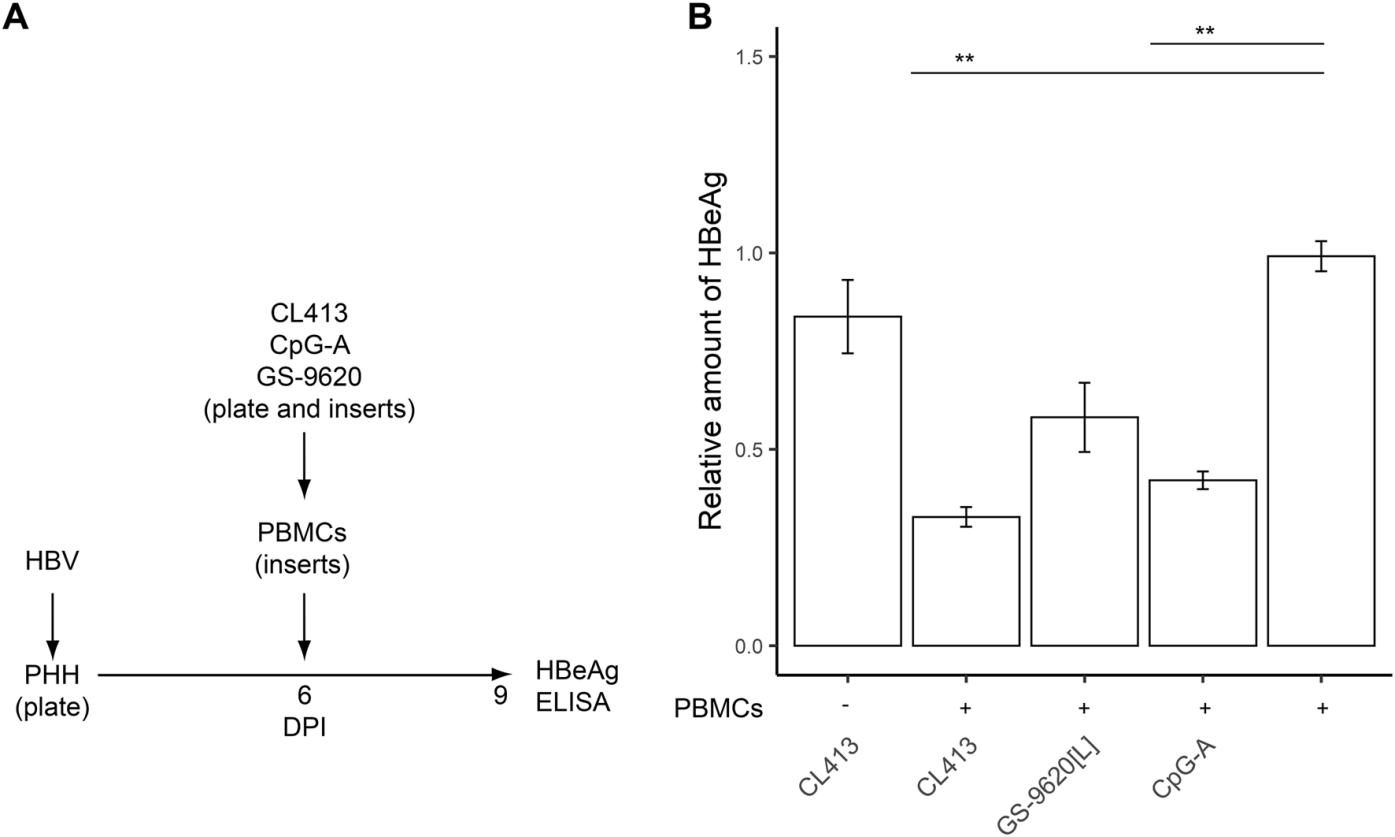
Inhibition of HBeAg production from HBV-infected PHH by coculture with TLR2/7, TLR7 or TLR9 agonist-stimulated PBMCs. (**A**) Experimental flow chart. PHH were infected with HBV and kept in culture for 6 days before a Transwell insert containing PBMCs stimulated with the TLR2/7 agonist CL413 (5 µg/ml), TLR9 agonist CpG-A (4 µg/ml), or TLR7 agonist GS-9620[L] (50 nM) was added. (**B**) Production of HBeAg was normalized to production of HBV-infected PHH cells cocultured with non-stimulated PBMCs. In one parallel, a culture of HBV-infected PHH was exposed to CL413 in the absence of PBMCs. Data are shown as mean ± SEM of three biological replicates with PHH from two donors (N = 2). **p < 0.01, Mann–Whitney–Wilcoxon pairwise test, *p* value adjusted by BH method.

**Coculturing with stimulated PBMCs inhibits HBV production from PHH.** To test whether continuous production of cytokines from TLR2/7, TLR7, or TLR9 agonist-stimulated PBMCs inhibits the production of HBeAg from HBV-infected PHH more strongly than two-times addition of CM to infected cells, we cocultured TLR agonist-stimulated PBMCs with HBV-infected PHH in the Transwell system from 6–9 DPI (Fig. 4A). We found that inhibition of HBeAg in HBV-infected PHH by coculture with TLR2/7 (CL413), TLR7 (GS-9620[L]) or TLR9 (CpG-A) agonist-stimulated PBMCs did not significantly differ from inhibition following two-times addition of CM (Fig. 4B). In any case, inhibition did not exceed 70%, and CL413 was a more potent inducer of an antiviral response than CpG-A or GS-9620[L]. Previous study found that several TLR agonists can inhibit HBV replication both directly via TLR activation in PHH and indirectly via exposure to CM of stimulated innate immune cells^23^. Thus, we tested whether the TLR2/7 dual agonist CL413 can inhibit HBV replication without the indirect effect of PBMC-secreted cytokines (Fig. 4B). However, in the absence of PBMCs, CL413 did not show any antiviral activity, although it induced production of proinflammatory cytokines IL-6 (275 pg/ml), TNF-α (84 pg/ml) and chemokine IL-8 (987 pg/ml) in HBV-infected PHH. As in the case of CM addition to HBV-infected cells, coculture of TLR agonist-stimulated PBMCs with HBV-infected PHH in the Transwell system did not result in degradation of cccDNA (data not shown).

**Figure 5:**
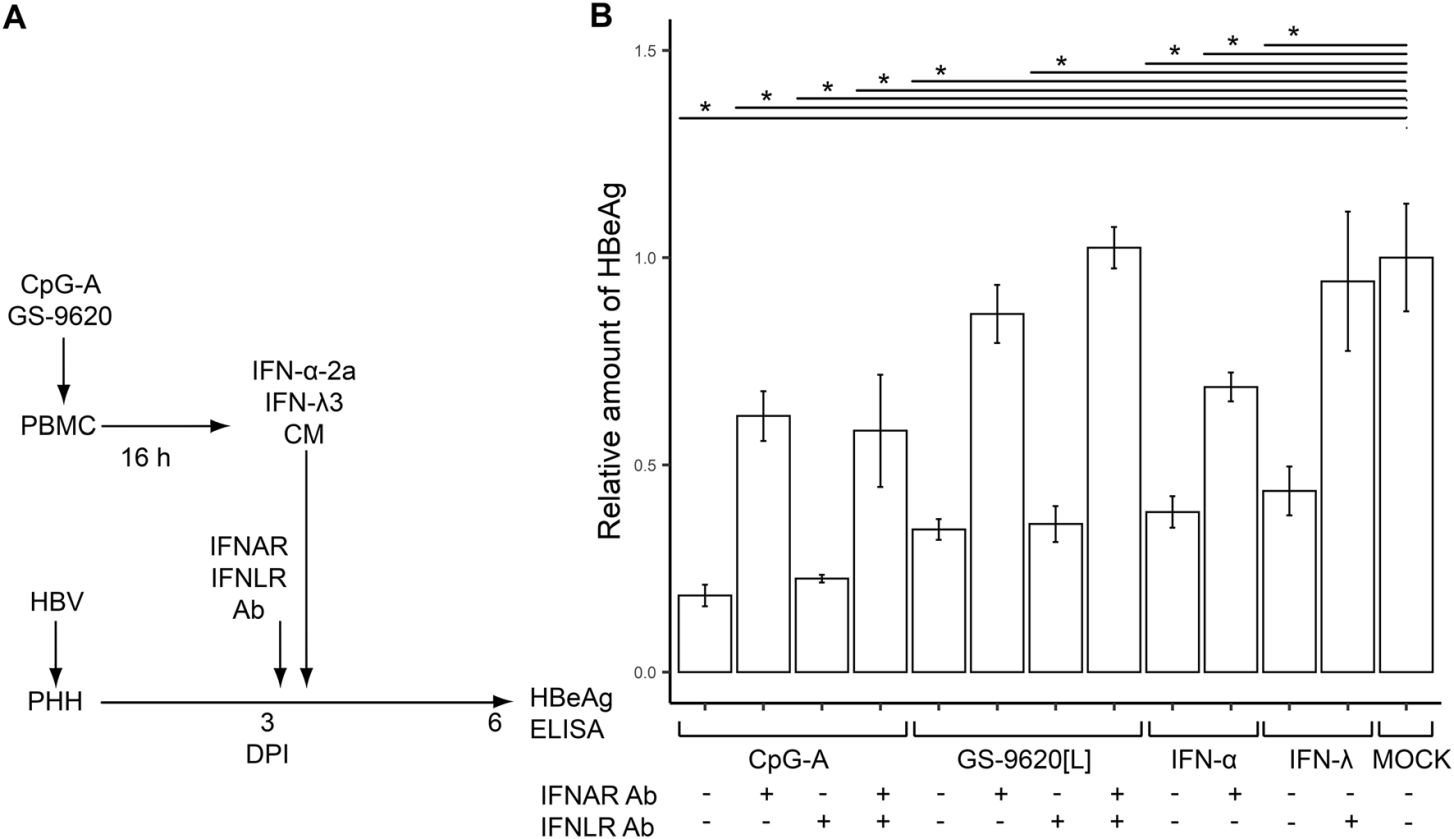
IFNAR mAb abrogates inhibition of HBeAg production from HBV-infected PHH by CpG-A-CM or GS-9620[L]-CM. (**A**) Experimental flow chart. HBV-infected PHH were exposed at 3 DPI to 5 µg/ml of IFNAR mAb, IFNLR mAb or control isotype mAb. Recombinant IFN-α-2a or IFN-λ3 (1,000 IU/ml) was used as a control. Production of HBeAg was determined by ELISA 6 DPI. (**B**) Production of HBeAg was normalized to production by HBV-infected PHH treated with CM from unstimulated PBMCs determined by ELISA 6 DPI. The data are shown as mean ± SEM from three independent experiments with PHH from one donor (N = 1). *p < 0.05, Mann–Whitney–Wilcoxon pairwise test, *p* value adjusted by BH method.

**Anti-IFN-I receptor monoclonal antibody (IFNAR mAb) abrogates CpG-A-CM or GS-9620[L]-CM-induced inhibition of HBeAg production from HBV-infected PHH.** Subsequently, we investigated the mechanism of pDC-induced inhibition of HBeAg production in HBV-infected PHH. Due to the sensitivity of HBeAg production in HBV-infected PHH to IFN-α and IFN-λ, we examined the proportion of the inhibitory effect mediated by type I and III IFN receptors, IFNAR and IFNLR (Fig. 5A). To do so, we pretreated HBV-infected PHH with mAbs targeting IFNAR and IFNLR and determined the level of HBeAg produced upon exposure of HBV-infected PHH to CpG-A-CM or GS-9620[L]-CM (Fig. 5B). While IFNAR mAb completely abrogated the inhibitory effect of GS-9620-CM, it abrogated by only 40% the inhibitory effect of CpG-A-CM. Simultaneous blockade of IFNAR and IFNLR did not significantly increase abrogation of the inhibitory effect on HBeAg production. Production of HBeAg was also significantly inhibited by 1,000 IU of recombinant IFN-α2a (by 61.3%, *p* = 0.04) and IFN-λ3 (by 56.2%, *p* = 0.04).

**Figure 6:**
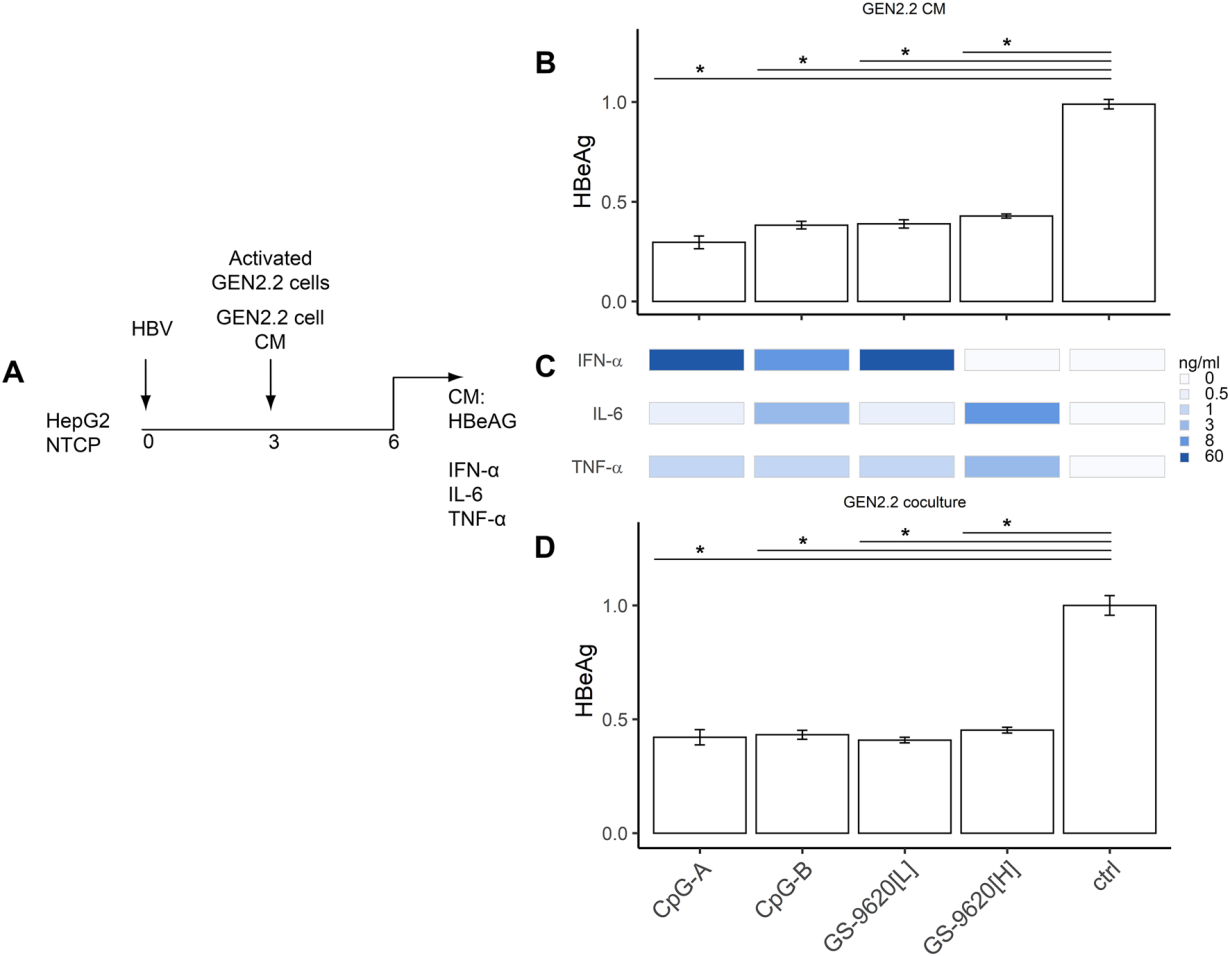
Antiviral activity of stimulated GEN2.2 pDCs on HBV-infected HepG2-NTCP cells. (**A**) Experimental flow chart. A total of 60,000 HepG2-NTCP cells were infected with 2000 VGE per cell of HBV and cultured for 3 days before CM from GEN2.2 cells stimulated with CpG-A (4 µg/ml), CpG-B (4 µg/ml), or GS-9620[L or H] (50 nM or 10 µM) was added (**B**). Levels of IFN-α, IL-6 and TNF-α (**D**) in CM were determined by ELISA 6 DPI. HBV-infected HepG2-NTCP cells were cocultured with 100,000 stimulated GEN2.2 cells (**C**). Production of HBeAg was normalized to production by HBV-infected HepG2-NTCP cells in the absence of pDCs. The data are shown as means ± SEM from three independent experiments. *p < 0.05, Mann–Whitney–Wilcoxon pairwise test, *p* value adjusted by BH method.

**Stimulated GEN2.2 pDCs show antiviral activity against HBV-infected HepG2-NTCP cells. **We next compared the antiviral effect of CM from stimulated PBMCs on virus production in PHH (Fig. 2) with that in a model comprising GEN2.2 pDCs and HBV-infected HepG2-NTCP hepatocytes^26,38,39^ (Fig. 6A). To facilitate 4 days lasting coculture, which is still difficult to perform in rare and in vitro short living human primary pDCs, we performed our studies in human pDC line GEN2.2, which shares many features with human primary pDCs^26,39,40^. Production of HBeAg from HBV-infected HepG2-NTCP hepatocytes was significantly inhibited by exposure to CM from GEN2.2 cells stimulated with CpG-A (by 67%), CpG-B (by 59%), GS9620[L] (by 55%) and GS-9620[H] (by 53%) (Fig. 6B). No significant differences in inhibition of HBeAg production were observed when the antiviral effect of CM from stimulated GEN2.2 cells was compared with direct coculture of GEN2.2 and HepG2-NTCP cells (Fig. 6C). Despite the different repertoires and levels of cytokines induced by CpG-A (50 ng/ml IFN-α), CpG-B (4 ng/ml IFN-α), GS-9620[L] (50 ng/ml IFN-α), and namely GS-9620[H] (< 50 pg/ml IFN-α) (Fig. 6D), all three agonists similarly inhibited HBeAg (by approximately 65%) (Fig. 6B,C).

## Discussion

In this study, we investigated the antiviral effect of CM from PBMCs stimulated with a set of agonists of endosome-localized TLRs. Our results demonstrate that synthetic TLR agonists capable of activating more than one TLR induce a broader proinflammatory cytokine spectrum and are more efficient drivers of HBV inhibition than single TLR-targeting agonists. The dual-acting TLR agonists R848^41^, CL413^35^ and GS-9620[H]^36^ were the best-scoring inducers of HBV inhibition. Statistical significance was a major issue in these experiments, which were performed in fresh PHH from 3 donors, each tested in 5 biological replicates. None of the selected TLR ligands reduced the level of cccDNA in HBV-infected cells. Previous findings revealed that R848 can be independently recognized by both human TLR7 and TLR8, although TLR8 is induced more efficiently than TLR7 at higher R848 concentrations^41^. A low concentration of GS-9620[L] has approximately 30-fold higher selectivity for activation of TLR7 over TLR8, with no detectable activity on other human TLRs^36^. However, GS-9620 elicits combined TLR7 and TLR8 stimulation at higher concentrations^36^. As TLR7 expression is largely restricted to pDCs in PBMC subsets^26–30,42^ and TLR8 is predominantly expressed in myeloid DCs and monocytes^43,44^, we surmise that low concentrations of GS-9620[L] (50 nM) preferentially mediate secretion of IFN-α from pDCs, whereas high concentrations of GS-9620[H] (10 µM) preferentially stimulate secretion of inflammatory cytokines in classical myeloid DCs and CD14+ monocytes.

Another dual-acting agonist, CL413, also induces both IFN-I and proinflammatory cytokines. We tested CL413 activity to analyze both a direct effect on TLR2 stimulation in PHH and an indirect effect on TLR7 stimulation in PBMCs. CL413 did not elicit a direct inhibitory effect on HBV replication in TLR2-expressing PHH. Therefore, its antiviral effect likely is conferred indirectly by triggering TLR2 and TLR7 in PBMCs. Expression of cytoplasmic TLR2 and endosome-localized TLR7 in CD14+ monocytes permits both signaling pathways to be triggered at the single-cell level^44^. In contrast, in pDCs in which TLR2 is not expressed, only TLR7 signaling can be activated by CL413. Recently, the dual-acting agonist Riboxxol, which triggers TLR2/3-mediated signaling via the IFN or NF-κB pathways, was shown to efficiently and directly suppress HBV replication in HBV-infected PHH^23^. In contrast to dual-acting agonists, single-acting ligands of TLR7 (CL264) or TLR9 (CpG-B) induced in PBMCs only moderate levels of proinflammatory cytokines with no detectable IFN-I, and CL264-CM and CpG-B-CM were associated with poor HBeAg inhibition. Robust production of proinflammatory cytokines induced in PBMCs by dual-acting agonists was associated with elevated phosphorylation of p65 NF-ĸB.

To induce a large and variable range of cytokines, we stimulated PBMCs with a larger spectrum of agonists than those used in previous studies^23,24^. Our results indicate that inhibition of HBeAg and HBsAg production in HBV-infected PHH does not correlate with the quantity of PBMC-secreted IFN-α, but rather is a complex function of multiple secreted cytokines. We found that CpG-A-activated PBMCs produced more IFN-I and IFN-III than those stimulated with GS-9620[L], which has been tested extensively in previous HBV inhibition-related studies^20–24^. In agreement with the previous finding that IFN-I is the major component of PBMC CM responsible for inhibition of HBV production in PHH, CpG-A-CM inhibited HBeAg and HBsAg secretion more efficiently than GS-9620[L]. However, a higher concentration of GS-9620[H] (10 µM), which induced in PBMCs a broader spectrum of proinflammatory cytokines but a lower quantity of IFN-I than induced by GS-9620[L], was associated with significantly higher HBeAg and HBsAg inhibition. R848, which also induced a very broad spectrum of proinflammatory cytokines, had a similar antiviral effect as GS-9620[H]. Importance of the cytokine complexity in inhibition of HBV production is further highlighted by relatively low inhibitory activity (50 to 60%) of recombinant IFN-α-2a and IFN-λ3. Based on the blockade of IFNAR by mAb, Lucifora et al.^23^ concluded that the antiviral effect of TLR1/2 and TLR3 activation in PHH was not due to type-I IFN and IL-6 production. Correlation analysis showed that in addition to IFN-α, the proinflammatory cytokines IL-6, TNF-α and IL-12 and the chemokines IL-8 and IFN-γ are major contributors to anti-HBV inhibitory activity. A statistical model that could decipher the specific combination of cytokines necessary to inhibit HBV production from infected hepatocytes would require additional measurements of the effect of CM or artificial permutations of recombinant cytokines.

Although R848-CM, CL-413-CM, CpG-A-CM and GS-9620[H]-CM achieved two to fourfold greater inhibition of HBeAg than that observed with GS-9620[L]-CM in previous studies^23,24^, none resulted in reduction of cccDNA levels in freshly isolated PHH. This is compatible with recent findings showing that GS-9620[L]-CM strongly induces various IFN-stimulated genes and inhibits virus production in HBV-infected PHH without inducing APOBEC3A or the Smc5/6 complex—and without reducing cccDNA levels^24^. The importance of cccDNA and its degradation for HBV cure positions this molecule in the center of HBV research. A 2012 study reported that IFN-α inhibits cccDNA transcription by hypoacetylation of cccDNA-bound histones and reduces binding of the STAT1 and STAT2 transcription factors to the IFN-stimulated response element present in the HBV genome^8^. More recent studies have shown that cccDNA can be degraded in HBV-infected hepatocytes in a noncytopathic fashion during IFN-α treatment^9,10^.

Production of HBeAg from the HBV-infected hepatoma cell line HepG2-NTCP was three- to fivefold less sensitive to IFN-α compared to production from HBV-infected PHH. In the presence of CM or recombinant IFN-I, residual production of HBeAg in HepG2-NTCP cells was not suppressed below 35%. The insignificant differences in inhibition of HBV production by exposure of HBV-infected HepG2-NTCP cells directly to activated GEN2.2 cells or to their CM indicate that soluble factors, and not cell-to-cell contact during coculture, plays a major role in the regulation of HBV production.

We also addressed whether IFN-I and IFN-III present in CpG-A-CM cooperate in HBV inhibition, which could explain the greater inhibitory effect of CpG-A-CM compared to GS-9620[L]-CM. Surprisingly, IFNLR targeting had no effect on HBeAg secretion, and only IFNAR mAb partially abrogated the inhibitory effect of CpG-A-CM. This inhibitory effect was not completely reverted by targeting both IFNAR and IFNLR, likely due to inefficient inhibition of IFNAR. Thus, we cannot conclude whether IFN-I is the main inhibitory driver in CpG-A-CM or if other cytokines contribute as well. Further study will be necessary to elucidate whether IFN-I signaling dominates over IFN-III signaling in PHH.

Specific and prolonged suppression of chronic hepatitis B in chimpanzee and woodchuck models by endosomal TLR7, 8, and 9 agonists led to an interest in discerning the mechanisms by which these TLR ligands elicit antiviral responses^20–22^. However, clinical studies with GS-9620, which showed the best antiviral effect in animal models, did not reveal a clinically significant decline of HBsAg at tolerable doses (two one-weekly doses 4 mg) in patients with chronic hepatitis B^45,46^. This dose corresponds to concentration of 4.6 ng/ml in plasma, while 50 ng/ml of GS-9620[L] were used in our experiments^47^. The dose of CpG ODNs commonly used in clinical trials was 1.5–15 μg/kg and the schedule of ODN administration ranged from weekly to monthly. In preclinical studies, much higher doses of CpG ODN (2.5 mg/kg) were administered daily to mice while 4 μg/ml of CpG-A or CpG-B were used in our experiments^48^. Also the dose of CL413 agonist (5 μg/ml) was within the range of the dose administered to mice in preclinical studies (3 μg/g)^49^. Further studies of the distinct antiviral potential of poly-specific TLR agonists are necessary to elucidate their functions, which could provide new opportunities for the development of novel strategies to achieve sustained viral clearance and provide a definitive cure for hepatitis B.

## Methods

### Hepatocyte cell cultures

HepG2-NTCP (a human liver cancer cell line, HepG2, stably transfected with the human HBV entry receptor—sodium taurocholate cotransporting polypeptide [hNTCP]) was obtained from Dr. Stephan Urban of Heidelberg University Hospital, Heidelberg, Germany. HepG2.2.15 (a HepG2 cell line that harbors two head-to-tail dimers of the HBV genome [serotype ayw, genotype D; GenBank accession: U95551.1]) was obtained from Dr. David Durantel of the Cancer Research Center of Lyon, Lyon, France. These cell lines were grown in Dulbecco’s modified Eagle’s medium supplemented with 10% fetal bovine serum (FBS) and puromycin (0.05 mg/ml) or G418 (0.4 mg/ml), respectively^50^. HepAD38 cells were maintained in Dulbecco’s modified Eagle’s medium supplemented with 10% FBS and tetracycline (0.3 µg/ml). Primary human hepatocytes (PHHs) were isolated from liver resections as described by David et al.^51^. Briefly, liver biopsies were first perfused with Hanks Balanced Salt Solution (HBSS) lacking Ca^2+^ and supplemented with 0.5 mM EGTA (Merck). Then, the liver tissue fragments were perfused with HBSS supplemented with Ca^2+^ and 0.05% Collagenase (Merck). The liver cell suspension was filtered through a 100 µm Cell Strainer (Corning), centrifuged at 50 × g for 3 min at 4 °C and washed 3 times with L-15 Medium (Thermo Fischer Scientific). Cell viability was estimated by trypan-blue exclusion, and the cells were seeded on collagen-coated plates. PHHs were maintained in Williams E Medium (Thermo Fisher Scientific) supplemented with the Primary Hepatocyte Maintenance Supplement Kit (Gibco).

### PBMCs and GEN2.2 cell line

PBMCs were isolated and cultured as previously described^37,52^. The GEN2.2 cell line was cultured with mouse MS5 cell line in RPMI 1,640 supplemented with 10% FBS^40^.

### Inhibitors, antibodies and reagents

CpG-A (ODN 2,216), CpG-B (ODN 2006), CL413 (Adilipolin), CL267 and R848 were obtained from InvivoGen (San Diego, USA) for use in in vitro PBMC stimulation assays, and GS-9620 was a gift from Gilead Sciences. All of them were used at concentration recommended by manufacturer for optimal in vitro stimulation. Recombinant IFN-α-2a and IFN-λ3 were obtained from PBL. Anti-Human Interferon Lambda Receptor 1 (IFNLR), clone MMHLR-1, neutralizing (MAb) was from PBL; Anti-IFN-α/β Receptor Chain 2 Antibody, clone MMHAR-2, MAB1155 was from EMD Millipore.

### Preparation of HBV

Two HepG2-derived cell lines were used for HBV production and purification: the 2.2.15 cell line and AD38 cell line. Infectious particles (Dane particles) were purified by 6% PEG-precipitation and centrifugation from collected cell-free supernatants.

### HBV infection of HepG2-NTCP cells and PHHs

HepG2-NTCP cells were infected with HepG2.2.15-derived HBV (2000 viral genome equivalents per cell) overnight in the presence of 4% PEG8000 and 2.5% DMSO. Then, HepG2-NTCP cells were washed 3 times with PBS and maintained in Dulbecco’s modified Eagle’s medium supplemented with 10% FBS and 2.5% DMSO. PHHs were infected with HepAD38-derived HBV (500 viral genome equivalents per cell) overnight in the presence of 4% PEG8000. Then, PHHs were washed 3 times with Williams E Medium (Thermo Fisher Scientific) and maintained in Williams E medium supplemented with the Primary Hepatocyte Maintenance Supplement kit (Gibco) and 2% DMSO.

### Detection of HBsAg and HBeAg secretion by ELISA

Cell-free supernatants from HBV-infected HepG2-NTCP cells or PHHs were collected and centrifuged at 300 × *g* for 5 min to remove cellular debris, transferred into clean tubes and stored at − 80 °C until antigen measurement. The titers of HBsAg and HBeAg were measured using a commercial ELISA kit (Bioneovan, Beijing, China) according to the manufacturer’s instructions.

### In vitro GEN2.2 and PBMC stimulation

To determine cytokine production, PBMCs (3 × 10^6^ cells/ml) or GEN2.2 (1 × 10^6^ cells/ml) were stimulated with CpG-A (4 µg/ml), CpG-B (4 µg/ml), CL413 (4 µg/ml), CL267 (4 µg/ml), R848 (4 µg/ml), and GS-9620 (50 nM or 10 µM) overnight.

### Total HBV DNA and cccDNA quantification

Total cellular DNA was isolated from HBV-infected PHHs with the NucleoSpin Tissue Kit (Macherey–Nagel). The total HBV DNA level was determined by quantitative PCR (qPCR) using primers specific for HBV DNA: HBV-F, 5′-AGAGGACTCTTGGACTCTCTGC-3′; HBV-R, 5′-CTCCCAGTCTTTAAACAAACAGTC-3′; and the probe pHBV, 5′-[FAM]TCAACGACCGACCTT[BHQ1]-3′. qPCR was performed with gb Elite PCR Master Mix (Generi Biotech) and TaqMan probe. The level of HBV DNA was normalized to albumin (Alb-F, 5′-GCTGTCATCTCTTGTGGGCTGT-3′; Alb-R, 5′-AAACTCATGGGAGCTGCTGGTT-3′; and Alb-probe, 5′-[FAM]GGAGAGATTTGTGTGGGCATGACAGG[BHQ1]-3′). cccDNA quantification was performed as previously described^50^. Briefly, 1 µg of DNA was treated with 10 units of T5 exonuclease for 2 h. Then, DNA was purified using a DNA Clean and Concentrator Kit (Zymo Research). qPCR was performed with gb Elite PCR Master Mix (Generi Biotech) and specific cccDNA primers and probe. The level of cccDNA was normalized to mitochondrial-encoded cytochrome-c oxidase subunit II (MT-CO2) expression in samples without T5 exonuclease digestion. cccDNA-specific primers and MC-CO2-specific primers were used as previously described^24^. ddPCR was performed using a QX200 Digital PCR Generator and QX200 Droplet Reader (both Biorad) with cccDNA-specific primers and probe. T5 treatment and cccDNA primer specificity was confirmed by comparing the T5-treated and non-treated samples and by comparing cccDNA specific primers with total HBV DNA primers (non-specific cccDNA primers).

### Blockade of IFNAR and IFNLAR

IFNAR was blocked by Anti-IFN-α/β Receptor Chain 2 Antibody, clone MMHAR-2, MAB1155 EMD (Millipore) at 5 µg/ml. IFN-λ receptor 1 was blocked by Anti-Human Interferon Lambda Receptor 1, clone MMHLR-1 (PBL) at 5 µg/ml. Mouse IgG1 control from murine myeloma clone MOPC 21 and mouse IgG2a isotype control from murine myeloma clone UPC-10 were used as isotype controls.

### Determination of secreted cytokines and chemokines

The quantities of total IFN-α, IFN-γ, IFN-λ1, TNF-α, IL-6, IL-12, IL-8 and IL-10 produced by PBMCs or GEN2.2 were measured in cell-free supernatants after 16 to 20 h culture using Human ELISA Kits (Mabtech).

### Statistical analysis

Quantitative variables are expressed as means ± standard error of the mean (SEM). Non-parametric tests were performed due to the nature of the data. First, Kruskal–Wallis was applied, followed by non-parametric post hoc pairwise multiple comparison Mann–Whitney–Wilcoxon test with *p* value adjustment by Benjamini Hochberg method (BH). In case of data depicted in Fig. 3^,^ Dunn’s test was performed also with *p* value adjustment by Benjamini Hochberg method (BH). All tests and pictures were computed and rendered in the R software package, figures were finalized in Adobe Photoshop CS. A *p* value ≤ 0.05 was considered significant.

### Ethics statement

This study was conducted according to the principles expressed in the Declaration of Helsinki. The study was performed according to local ethical regulations, following approval by the institutional ethics committee (review board) of the Institute of Experimental Medicine and Thomayer Hospital on March 9, 2016 (docket no. 363116 [G-16–03-02]). All liver tissue donors and PBMC donors provided written informed consent for participation in the study in accordance with institutional and regulatory guidelines.

## Supplementary information

Supplementary file1
