# Supplementary material for: Toll-like receptor dual-acting agonists are potent inducers of PBMC-produced cytokines that inhibit hepatitis B virus production in primary human hepatocytes
Source: Sci Rep. 2020 Jul 29;10:12767. doi: 10.1038/s41598-020-69614-7 (PMC7392756; doi:10.1038/s41598-020-69614-7)
Supplement: Supplementary file 1 — Supplementary file1 [file 41598_2020_69614_MOESM1_ESM.pdf]

SUPPLEMENTARY MATERIALS

Toll-like receptor dual-acting agonists are potent inducers of PBMC-produced cytokines that inhibit hepatitis B virus production in primary human hepatocytes

Vaclav Janovec[1,2], Jan Hodek[2], Kamila Clarova[2], Tomas Hofman[1,2], Pavel Dostalík[1], Jiri Fronek[3,4], Jaroslav Chlupac[3,4], Laurence Chaperot[5], Sarah Durand[6], Thomas F. Baumert[6,7], Iva Pichova[2], Barbora Lubyova[2], Ivan Hirsch[1,2,8†*], Jan Weber[2†*]

[1] Department of Genetics and Microbiology, Charles University, Faculty of Science, BIOCEV, 25150 Vestec, Czech Republic;
[2]Institute of Organic Chemistry and Biochemistry of the Czech Academy of Science, IOCB & Gilead Research Center, 16610 Prague, Czech Republic;
[3]Transplantation Surgery Department, Institute for Clinical and Experimental Medicine, 14021 Prague;
[4]Department of Anatomy, Second Faculty of Medicine, Charles University, 15006 Prague;
[5]Université Grenoble Alpes, CNRS UMR5309, Inserm U1209, CHU Grenoble Alpes, IAB, EFS, 38000 Grenoble, France;
[6]Universite de Strasbourg, Inserm, Institut de Recherche sur les Maladies Virales et Hepatiques UMRS 1110, F-67000 Strasbourg, France;
[7]Pole Hepato-Digestif, Institut Hospitalo-Universitaire, Hopitaux Universitaires de Strasbourg, F-67000 Strasbourg, France;
[8]Institute of Molecular Genetics of the Czech Academy of Sciences, 14220 Prague

[†]equal contribution

***Correspondence:**
Dr. Ivan Hirsch, Department of Genetics and Microbiology, Charles University, Faculty of Science, BIOCEV, 25150 Vestec, Czech Republic Tel.: +(420) 221951723, E-mail; hirschi@natur.cuni.cz;
Dr. Jan Weber, Institute of Organic Chemistry and Biochemistry of the Czech Academy of Science, IOCB & Gilead Research Center, 16610 Prague, Czech Republic. Tel.: +(420) 220183420, E-mail: weber@uochb.cas.cz

Supplementary Methods

Determination of NF-κB p65 phosphorylation by dynamic phospho-flow cytometry. PBMCs stimulated for 1 h with TLR agonists were fixed in 4% formaldehyde for 15 min, permeabilized by 90% methanol for 30 min, and incubated with Phospho-NF-κB p65 (Ser536) (93H1) rabbit mAb (Cell Signaling, Danvers, MA). APC-conjugated Goat anti-Rabbit IgG polyclonal antibody (Thermo Fisher Scientific) was used as a secondary antibody. Viable PBMCs were gated according to Live/Dead Zombie Green kit. Samples were analyzed using a BD LSR FORTESSA cytometer (BD Biosciences, San Jose, USA) and data were processed using FLOWJO software (Treestar, San Carlos, USA).

XTT test. Cytotoxic effect was analyzed by XTT colorimetric assay. Briefly, 50 µl of 50:1 mixture of XTT labeling reagent (1 mg/ml; Sigma-Aldrich, Life Science) and PMS electron-coupling reagent (0.383 mg/ml; Sigma-Aldrich, Life Science) was added to the wells with HBV-infected PHH and incubated for 4 h in 37 °C in 5 % CO_2. Formation of orange formazan dye was measured in Victor X3 plate reader.

Supplementary Results

Differential potencies of TLR2/7, TLR7, TLR7/8 and TLR9 agonists to induce repertoire of cytokines secreted by stimulated PBMCs.

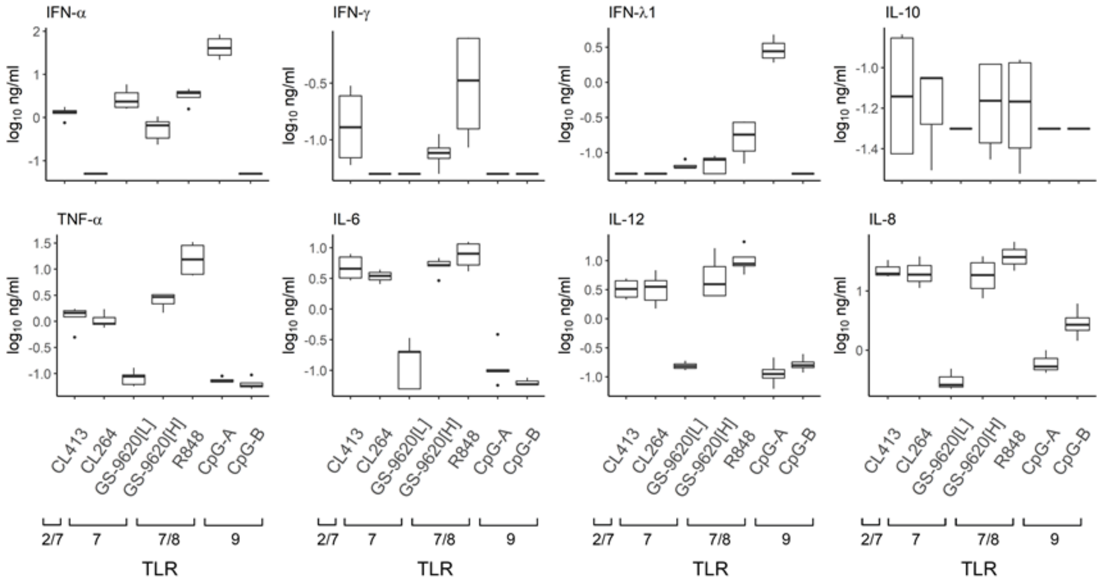

Figure S1. Amounts of cytokines secreted by PBMCs stimulated by different TLR2/7, TLR7, TLR7/8 and TLR9 agonists. PBMCs (N>3) were stimulated with either no agonist, the dual agonist CL413 (5 µg/ml), the TLR7 agonists CL264 (5 µg/ml) or GS9620[L], the TLR7/8 agonists GS9620[H] or R848 (4 µg/ml), or the TLR9 agonists CpG-A (4 µg/ml) or CpG-B (4 µg/ml) for 16 h, and the cytokine levels were determined by ELISA.

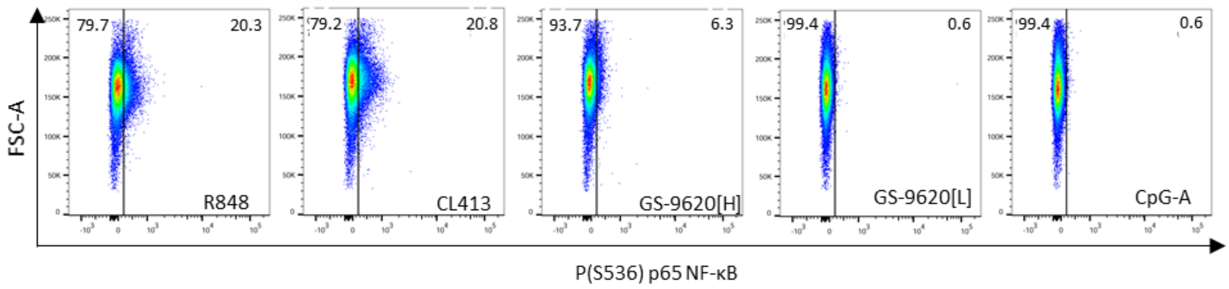

Figure S2. PBMCs were stimulated with the dual-acting agonists R848 (4 µg/ml), CL413 (5 µg/ml) and GS9620[H], or the single-acting agonists of TLR7, GS9620[L], or of TLR9, CpG-A (4 µg/ml), for 1 h, and the expression levels of P(S536) p65 NF-κB cytokine were determined by phospho-flow cytometry. Distribution of phosphorylated p65 NF-κB is shown as a percentage in the upper half of dot-plot diagrams.

Inhibition of HBsAg production from infected PHH exposed to CM from PBMCs stimulated by TLR2/7, TLR7, TLR7/8 and TLR9 agonists

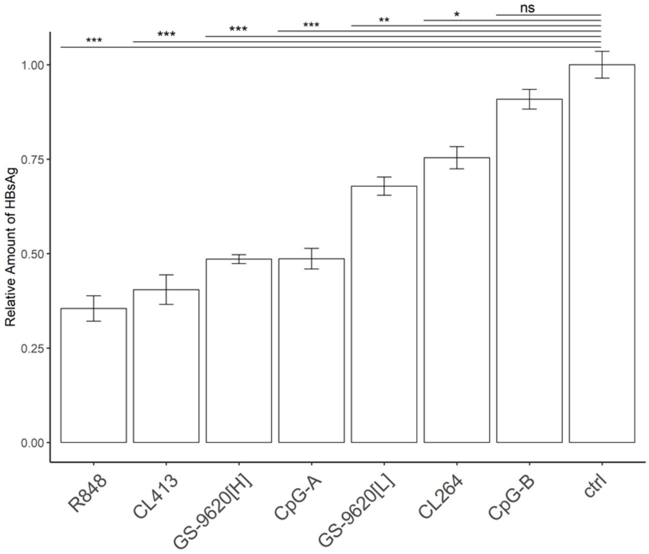

Figure S3. Inhibition of HBsAg production from HBV-infected PHH exposed to PBMC CM. A total of 30,000 PHH were infected with 2000 VGE of HBV and cultured for 3 days before CM (diluted 1:100) from 3×10^{6} PBMCs per ml stimulated by agonists of TLR2/7 (CL413), TLR7 (CL264, GS-9620[L]), TLR7/8 (R848, GS-9620[H]), TLR9 (CpG-A, CpG-B) was added. CM was added again 6 DPI. Production of HBeAg was determined by ELISA 9 DPI and normalized to production by HBV-infected PHH in the absence of CM. CM from unstimulated PBMCs was used as a control. Cells were grown for 3 more days. The HBsAg data are shown as mean ± SEM of 5 independent experiments with PHH from three donors. *, $p < 0.05$, **, $p < 0.01$, ***, $p < 0.001$ pairwise Post hoc Mann-Whitney-Wilcox**on** test. Kruskal-Wallis $p<3.6\times e^{-16}$.

Table S1. Viability of PHH treated with PBMC CM[1)]

TLR agonist	R848	CL413	GS-9620[H]	CpG-A	GS-9620[L]	CL264	CpG-B	Ctrl
Viability (%)	106.4±7.6	104.9±4.1	114.0±4.1	95.5±2,5	97.0±2.4	87.3±1.4	97.2±1.5	100±4.5

[1)]Determined by XTT test. Conditioned medium (CM) diluted 1:10.

Table S2. Multivariate statistics for HBeAg measurements[1)]

	CL413	CL264	GS-9620[L]	GS-9620[H]	R848	CpG-A	CpG-B
CL413	-	-	-	-	-	-	-
CL264	5.70e-09	-	-	-	-	-	-
GS-9620[L]	5.70e-09	1.40e-07	-	-	-	-	-
GS-9620[H]	2.26e-02	5.70e-09	5.70e-09	-	-	-	-
R848	4.00e-07	5.70e-09	5.70e-09	6.70e-09	-	-	-
CpG-A	1.20e-07	5.70e-09	5.70e-09	2.40e-04	5.70e-09	-	-
CpG-B	9.10e-09	1.50e-08	7.10e-09	9.10e-09	7.10e-09	7.10e-09	-
Ctrl	5.70e-09	5.70e-09	5.70e-09	5.70e-09	5.70e-09	5.70e-09	2.24e-03

[1)]The Kruskal-Walis (p-value <2.2e-16) test indicates differences between groups of agonists. Post hoc Mann-Whitney-Wilcoxon test indicate that all groups of agonists differ.

Table S3. Correlation across PBMC-secreted cytokines[1)]

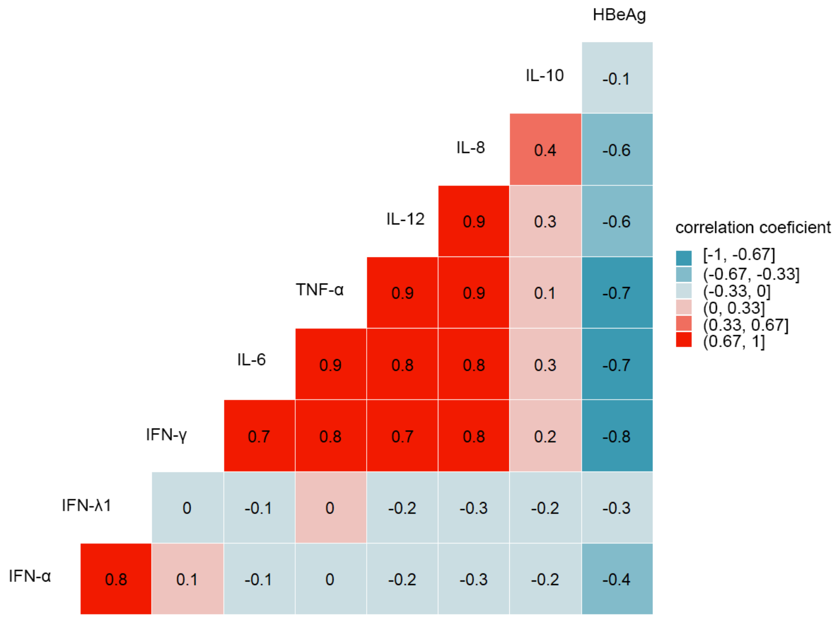

[1)]The data show correlation coefficients across PBMC-secreted cytokines and HBeAg produced from HBV-infected PHH.

Table S4. Determination of HBV cccDNA by droplet digital PCR

Sample	no T5 treatment		T5 treatment	
	cccDNA primer		cccDNA primer	
	non specific *(copies/μl)*	Specific *(copies/μl)*	non specific *(copies/μl)*	Specific *(copies/μl)*
GS-9620[H]/CM sample 1	7200	86.9	80.8	28.6
GS-9620[H]-CM sample 2	6130	75.5	46.5	26.4
R848-CM sample 1	7290	89.5	105	33.5
R848-CM sample 2	8400	101.5	110	37.4
CL413-CM	6560	78	80.7	32
CTRL-CM	< 10000	121	129	37.3
NO HBV	44.1	No Call	No Call	No Call
